# Editorial: Unveiling immunological mechanisms of periodontal diseases

**DOI:** 10.3389/fimmu.2022.1081158

**Published:** 2022-11-11

**Authors:** Teun J. de Vries, Pedro Paulo Chaves de Souza

**Affiliations:** ^1^Department of Periodontology, Academic Centre for Dentistry Amsterdam (ACTA), Amsterdam, Netherlands; ^2^The Innovation in Biomaterials Laboratory, School of Dentistry, Federal University of Goiás, Goiânia, Brazil

**Keywords:** periodontitis, osteoclast, inflammation, Th17, macrophages, osteoblasts, Porphyromonas gingivalis, gingivitis

Periodontal diseases, including periodontitis and gingivitis, are among the most common diseases in humans, afflicting 46% of adults aging 30 years or older in the United States between 2009-2012 (1). Gingivitis is usually harmless but may last long if left untreated. In this case, during the progression of gingivitis, the symbiotic biofilm suffers a shift in its composition and gradually, a dysbiotic condition between the microorganisms and the host may lead to the development of periodontitis. The severity of periodontitis will depend not only on the microorganisms, but also on the host immune response. In its advanced form, due to overactivation of the immunological system, periodontitis leads to periodontal ligament and alveolar bone destruction, sometimes resulting in tooth loss. Thus, understanding the host-pathogen interactions and the immunological mechanisms of periodontal disease is crucial for proposing new diagnostic and therapeutic approaches. This collection of 17 articles, written by 131 authors from 13 countries, has unveiled some of periodontitis’ immunological mechanisms.

## *Porphyromonas gingivalis*: cellular interaction with TLR2 and relation with Alzheimer’s disease

The association of *Porphyromonas gingivalis* (Pg) with periodontitis, as contributing to the dysbiosis of the microbiome of the sulcus, has been known for decades. Interaction of pathogen-associated molecular patterns from periodontopathogens with osteoclasts and their precursors is known to modulate differentiation and resorptive activity (2). Thus, much research has been done to identify pathogen recognition patterns expressed by eukaryotic cells that recognize Pg. In the context of Pg’s LPS, TLR4 has been studied most widely. However, it has been known that Pg also interacts with TLR2. Wielento et al., making use of various strains of Pg, describe that the fimbriae as well as the peptidylarginine deiminase (PPAD) activity of Pg is required for TLR2 activation. Recent studies have revealed that Pg is present in brains of Alzheimer’s disease patients (3). In a quest for elucidating the effect of Pg in the brain on gene expression, Patel et al. have revealed brain areas that are more affected such as the hypothalamus, cholinergic neurons, and the basal forebrain. Since Pg sheds the proteolytic enzymes gingipains, needed for invasion, they next also assessed what candidate proteins are cleaved by gingipains based on the known structure.

## Novel biomarkers for periodontal disease and association of periodontitis with systemic diseases

Advances in systems biology and bioinformatic tools allowed the identification of novel biomarkers of periodontal disease and its association with systemic diseases. Using proteomics, Liu et al. nine differentially expressed proteins between healthy and inflamed gingiva that were all confirmed with Western blotting. Pan et al. have employed bioinformatics analysis to further elucidate the ferroptosis genes in existing datasets of periodontitis and Type 2 diabetes. Nolde et al. have used data from genome wide association studies demonstrate that depression and periodontitis do not share a common heritability or a causal connection.

## Clinical studies

Clinical studies, such as that performed by Kanjevac et al. demonstrating that dental caries affects periodontal expression of cytokines, are important to further understand the pathogenesis of periodontal disease. Alim et al. demonstrated that pleckstrin (PLEK) is a potential biomarker of periodontitis being expressed by gingival fibroblasts and present in the saliva of chronic periodontitis patients in a higher level than healthy controls.

## Th17 cells and IL-17

An observational study by Wang et al. revealed that Th17/Treg imbalance is associated with abnormal fatty acid and amino acids metabolism and impaired glycolysis in gingivitis patients. For those readers interested in delving deeper into the role of IL-17 in periodontitis, the review by Huang et al. provides an excellent summary of recent findings on the effects of IL-17 on periodontitis and its regulation by A20, an ubiquitin-editing enzyme with anti-inflammatory activity.

## Mouse periodontitis models unveil the role of specific macrophages and the inflammasome protein NRLP3

Animal models provide an excellent tool for the discovery of the role of new molecules, or new roles for old molecules, on periodontitis (de Vries et al.). In diabetic mice, Byun et al. identified an exosomal micro-RNA, miR25-3p that was found to interfere with CD69. Their data suggest that exosomal miR-25-3p in saliva contributes to development and progression of diabetes-associated periodontitis. Combination of animal models with recent techniques such as single cell RNAseq (scRNAseq) provides a powerful tool for identification of specific cell populations involved in physiopathological processes. Xu et al. used scRNAseq and employed pharmacological blockage of CCR2 to demonstrate that the CCR2+ macrophages population modulate bone modeling during orthodontic tooth movement in mice. The presence of CCR2+ macrophages was confirmed in human periodontal tissues after application of orthodontic force. Azevedo et al. investigated the role of VIP (Vasoactive intestinal peptide) and PACAP (Pituitary adenylate cyclase activating polypeptide), two molecules favoring M2 phenotype, on alveolar bone healing after tooth extraction in mice. Despite the significant immunomodulatory effect, VIP and PCAP did not change the bone healing outcome. For the readers interested in macrophage polarization during periodontitis, our collection brings a review by Sun et al. in this topic. Li et al. provide a state-of-the-art knowledge on what is known about inflammasomes and the ultimate expression of inflammatory cytokines of the cells of the periodontium. Clinical evidence in gingivitis shows upregulation of the widely studied inflammasome component NRLP3, that is connected to increased IL-1β. Surprisingly, mice lacking NRLP3 (Cheat et al.) develop less bone loss after ligature mediated inoculation of Pg, suggesting a regulatory pathway modulating periodontitis. Overexpression of NRLP3, however, led to IL-1β expression, neutrophil invasion and ultimately in osteoclast recruitment and bone loss.

From a mechanistic perspective, the use of isolated cells provides an excellent tool for the understanding cell response to external stimuli and the intracellular signaling pathways activated by different agonists. The study by Zhou et al. opens a new possibility for modulation of the inflammatory response by targeting the Taste receptor family 2 (TAS2Rs) in gingival fibroblasts to control inflammation in periodontitis.

## New roles for osteoblasts

When considering periodontitis, it is evident that cells lining the bone, such as osteoblasts must be zoomed in on, since they may play a role in activating osteoclasts, the degraders of bone. Probably due to the fact that osteoblastic cells from the alveolar bone are difficult to obtain as a pure source (4) most experimental work on the mesenchyme derived cells has been performed on gingival and periodontal ligament fibroblasts. Zhou and Graves summarize the effect on inflammatory factors and the activated immune system on osteoblasts lining the bone. They conclude that all the cells present in the periodontal tissues are likely to participate one way or the other in the development of gingival inflammation that can transition to periodontitis and loss of supporting bone for the teeth. Though likely with greatly differing efficiency, all cells of the periodontium probably interact with bacterial products. This leads to an involvement of inflammasomes, supramolecular protein complexes assembled in response to pattern recognition receptors and damage-associated molecular patterns, leading to the maturation and secretion of pro-inflammatory cytokines and activation of inflammatory responses.

## Unveiling continues!

When starting our series on Unveiling Immunological Mechanisms of Periodontal Diseases, we deliberately choose “Unveiling” rather than “Unraveling”. Unveiling has the beautiful connotation to it that a certain view of an object or a person, here periodontitis, is obscured to some extent, but with having some idea about the dimensions. When removing a veil, one gets a more complete understanding of the other properties of what has been hidden. Now, at the closure of the series, we can look back at an unexpected diversity of entries. Our collection of 17 articles has unveiled some of periodontitis’ immunological mechanisms. A second series will succeed this series, now named “*Community Series in Unveiling Immunological Mechanisms of Periodontal Diseases, volume II*” (link: https://www.frontiersin.org/research-topics/45704/community-series-in-unveiling-immunological-mechanisms-of-periodontal-diseases-volume-ii).

## Author contributions

All authors listed have made a substantial, direct, and intellectual contribution to the work and approved it for publication.

## Conflict of interest

The authors declare that the research was conducted in the absence of any commercial or financial relationships that could be construed as a potential conflict of interest.

## Publisher’s note

All claims expressed in this article are solely those of the authors and do not necessarily represent those of their affiliated organizations, or those of the publisher, the editors and the reviewers. Any product that may be evaluated in this article, or claim that may be made by its manufacturer, is not guaranteed or endorsed by the publisher.
